# Faculty Responsible for the Accreditation Process of Medical Education in Korea Should Have Their Workloads Reduced

**DOI:** 10.3352/jeehp.2009.6.1

**Published:** 2009-12-20

**Authors:** Sun Huh

**Affiliations:** Department of Parasitology, College of Medicine and Institute of Medical Education, Hallym University, Chuncheon, Korea.

In Korea, the year 2000 marked the official launch of the accreditation system for medical education. The first accreditation project covered 41 medical schools from 2000 to 2004. During the first project, 32 schools received full accreditation, while 9 schools received conditional accreditation since they were new medical schools. Beginning in 2007, the second round of accreditation began, and it will end in 2010. Recently, there has been discussion on how to use the results of accreditation and how to improve the accreditation system at a meeting of "the 5th Anniversary of the Korean Institute of Medical Education and Evaluation (KIMEE) & the 10th Anniversary of the Accreditation Board of Medical Education in Korea" in Seoul, November 18, 2009 [1]. "The proposed usage of results included benefits for accredited schools and limitations for failing accreditation. Accredited schools would received selective government support. Graduates from failed schools would be limited in internship, residency applications, medical specialist boards examination or application in the Korean Medical Licensing Examination. Eventually, their status would be announced and the school closed."

Also, the following plan of improvement was suggested: evaluation and reports by students, questionnaires to students at admission and graduation, reporting every two years, etc. Those suggestions for usage of results is not so easy at the current time since the laws must be revised and the authority of the accreditation body should develop wide recognition by the public. Meanwhile, the plan of improvement might be easier to realize during the next round of the accreditation process.

This accreditation system definitely has contributed to the successful development of medical education in Korea. However, I would like to suggest one more point of improvement during the accreditation process. The present peer review process puts an undue burden on the members of the medical faculty who take on the job of reporting. The work required for writing and interpreting the data is too extensive. The database system KOMSIS (Korea Medical School Information System) should be sufficient to provide the information necessary for accreditation. If KOMSIS contains enough information for peer review, the burden on responsible faculty members can be reduced. Furthermore, faculty research and publication productivity should be checked by the accreditation body by searching KoreaMed [2], PubMed, and Web of Science. That is the method used in other evaluation project for each university carried out by the mass media. Currently, the calculation of productivity through publication of scientific papers is too complex to be summarized. If not only data inputted to KOMSIS are reviewed but also scientific productivity is checked by the accreditation body, the work burden of responsible faculty would be diminished and taking on this job would be a more pleasant and rewarding experience.
